# Systematic 12- and 13-core transrectal ultrasound- or magnetic resonance imaging-guided biopsies significantly improve prostate cancer detection rate: A single-center 13-year experience

**DOI:** 10.3892/ol.2014.2353

**Published:** 2014-07-15

**Authors:** GONG CHENG, YUAN HUANG, BIANJIANG LIU, RUIZHE ZHAO, PENGFEI SHAO, JIE LI, CHAO QIN, LIXIN HUA, CHANGJUN YIN

**Affiliations:** Department of Urology, The First Affiliated Hospital of Nanjing Medical University, Nanjing, Jiangsu 210029, P.R. China

**Keywords:** prostate cancer, biopsy, transrectal ultrasound, magnetic resonance imaging

## Abstract

The aim of the present study was to evaluate the value of systematic 12- and 13-core biopsies, guided by transrectal ultrasound (TRUS) or magnetic resonance imaging (MRI), with regard to the prostate cancer detection rate (PCDR). Between July 1999 and June 2012, 2,707 patients were recruited to the Department of Urology, The First Affiliated Hospital of Nanjing Medical University (Nanjing, China). Prostate biopsies were performed via systematic 12- or 13-core biopsy and guided by either TRUS or MRI. The PCDR was established by retrospectively analyzing the distribution of positive cores, and it was assumed that all patients had undergone four biopsy schemes: Medial 6-core, lateral 6-core, 12-core and entire 13-core. In addition, the positive rate of the biopsies with the extra 13th core and the mean positive rate of systematic 12-core biopsies were compared. The PCDR of an entire 13-core biopsy was significantly higher than that of a lateral 6-core biopsy. The positive rate of the extra 13th core, which identified abnormal TRUS or MRI findings, was significantly higher when compared with that of the mean positive rate of the systematic 12-core biopsy. The results of the present study demonstrated that the entire 13-core biopsy was superior to the 6-core biopsy with regard to the PCDR. Therefore, the systematic 12-core biopsy with an extra 13th core is considered to be beneficial towards improving the PCDR.

## Introduction

The sextant method for performing a prostate biopsy was introduced by Hodge *et al* (1) in 1989. Various studies have demonstrated that extra cores improve the prostate cancer detection rate (PCDR) (2–7). In previous years, prostate biopsies were guided by the finger of the operator, however, transrectal ultrasound (TRUS) is a simple and useful tool that may be used for detecting and observing prostate tissues. In recent years, the TRUS-guided biopsy has generally been adopted when a prostate biopsy is required (1,8,9). Recently, magnetic resonance imaging (MRI)-guided and robotic-assisted prostate biopsies have been attempted at various advanced medical centers (10,11); however, TRUS-guided prostate biopsy remains the standard regimen for prostate cancer detection at the majority of medical centers. The optimal number of biopsy cores and distribution, however, remains controversial.

In the present study, the 13-year data of finger- and TRUS-guided biopsies, which were conducted at the Department of Urology, The First Affiliated Hospital of Nanjing Medical University (Nanjing, China) were retrospectively analyzed. The value of entire 13-core biopsy, guided by either TRUS or MRI, with regard to the PCDR was evaluated; particularly the extra 13th core, which revealed abnormal TRUS or MRI findings. To the best of our knowledge, it is the largest and longest single-center study regarding prostate biopsies in a Han Chinese population.

## Patients and methods

### Patients

Between July 1999 and June 2012, 2,707 patients from the Han Chinese population were recruited for a prostate biopsy at the Department of Urology, The First Affiliated Hospital of Nanjing Medical University. All patients underwent a digital rectal examination (DRE), serum prostate-specific antigen (PSA) and free PSA (fPSA) detection and TRUS to assess the prostate volume (PV) prior to the biopsy. PSA density (PSAD) was defined as the ratio of PSA to PV and the f/t ratio was calculated as fPSA divided by PSA. A finger-guided biopsy was performed on 1,603 patients prior to July 2009 and 1,104 patients underwent TRUS-guided biopsy after June 2009. In addition, 60 patients underwent prostate MRI as well as TRUS after March 2012.

Approval for the study was granted by the ethics committee of Nanjing Medical University and written informed consent was obtained from all patients.

### Biopsy procedures

**Figure 1:**
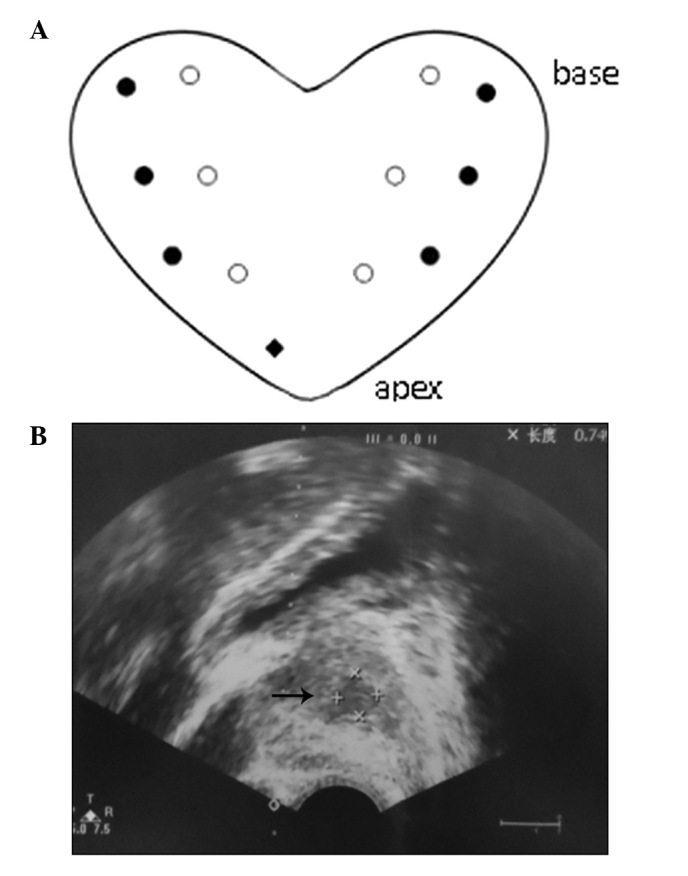
(A) Distribution of systematic 12-core biopsy. Empty circles, medial 6-cores; filled circles, lateral 6-cores; rhombus, extra 13th core in patients without abnormal transrectal ultrasound (TRUS) or magnetic resonance imaging findings. (B) A representative image of an abnormal TRUS. The arrow demonstrates the hypoechoic lesion.

The prostate biopsies were performed as systematic 12-core biopsies and for the TRUS-guided biopsy, an extra 13th core was added. The 12 cores were evenly distributed around four vertical planes: Right lateral, right medial, left medial and left lateral. Three biopsy cores from each plane were respectively located at the apex, middle and base of the prostate. The extra 13th core was directed towards the hypoechoic lesions on the TRUS image. In the patients that exhibited an abnormal MRI signal, the extra 13th core was directed towards the prostate area where the MRI demonstrated the lesions. For patients with normal TRUS and MRI images, the extra 13th core was positioned at the apex of the prostate (Fig. 1). The number and distribution of the positive cores were recorded. In order to perform further analyses, the distribution of positive cores were analyzed retrospectively, and it was assumed that all patients had undergone four biopsy schemes: Medial 6-core, lateral 6-core, 12-core and entire 13-core (Fig. 1). The data from each of these hypothetical biopsy schemes were compared. The positive rate of the 13th core was compared with the mean positive rate of a systematic 12-core biopsy (mean number of positive cores divided by 12) in patients with confirmed prostate cancer in order to evaluate the value of the extra 13th core.

### Statistical analysis

Data were expressed as the mean ± standard deviation and analyzed using SPSS software (version 18.0; SPSS Inc., Chicago, IL, USA). Differences between the PCa and non-PCa groups were assessed by the t-test and the χ^2^ test was used to compare nonparametric variables. P<0.05 was considered to indicate a statistically significant difference.

## Results

### Patient demographics and clinical characteristics

The demographic and clinical characteristics of 2,707 patients are presented in Table I. 36.2% (979/2,707) of the patients were confirmed with prostate cancer. The PCDR of the finger- and TRUS-guided biopsies was 32.5% (521/1,603; data not shown) and 41.5% (458/1,104), respectively.

### PCDR of the finger- and TRUS-guided biopsies

The PCDR of the finger- and TRUS-guided biopsies in different PSA and PV subgroups was further analyzed (Table II). In the patients with PSA ≤30 ng/ml or PV >46 cm^3^, the PCDR of the TRUS-guided biopsy was found to be significantly higher than that of the finger-guided biopsy (30.0% vs. 22.2%, P<0.001 and 31.7% vs. 18.1%, respectively). There was no statistical difference identified in the PCDR at PSA >30 ng/ml (79.1% vs. 73.4%, P=0.111) or PV ≤46 cm^3^ (46.8% vs. 44.3%, P=0.336).

### PCDR of various TRUS-guided biopsies

Table III shows the PCDR of the hypothetical medial 6-core, lateral 6-core, 12-core and entire 13-core TRUS-guided biopsies. The PCDR of the medial 6-core biopsy was identified to be significantly inferior to the lateral 6-core and 12-core biopsies (32.2% vs. 37.0%, P=0.020 and 32.2% vs. 40.7%, p<0.001, respectively). However, there was no obvious difference in the PCDR between the lateral 6-core and 12-core biopsies (37.0% vs. 40.7%, P=0.081). The PCDR of the entire 13-core biopsy was found to be significantly higher than the lateral 6-core biopsy (41.5% vs. 37.0%, P=0.033), although there was no obvious difference when compared with the 12-core biopsy (41.5% vs. 40.7%, P=0.729).

### Positive rate of the 13th core in TRUS- or MRI-guided biopsy

Table IV demonstrates the positive rate of the extra 13th core and the mean positive rate of the systematic 12-core in patients with confirmed prostate cancer, guided by TRUS or MRI. In 151 patients with hypoechoic lesions identified on the TRUS image, the positive rate of the extra 13th core was 70.9%. The 32 patients out of the total 60 who also underwent MRI exhibited abnormal signals, in which the positive rate of the extra 13th core was 81.2%. The mean number of positive cores in each patient undergoing TRUS-guided biopsy was 6.8, thus, the mean positive rate of the systematic 12-core biopsy was 56.7% (6.8/12). The positive rate of the extra 13th core, which was directed towards the abnormal TRUS or MRI findings, was found to be significantly higher than the mean positive rate of the systematic 12-core biopsy (70.9% vs. 56.6%, P<0.001 and 81.2% vs. 56.6%, P=0.006, respectively). Although the MRI-guided biopsy was associated with a higher PCDR than the TRUS-guided biopsy, the difference was not identified to be significant (81.2% vs. 70.9%, P=0.280).

## Discussion

The present study summarizes the 13-year experience of prostate biopsies on a large, Han Chinese population at the Department of Urology, The First Affiliated Hospital of Nanjing Medical University. TRUS-guided biopsies have been widely adopted in advanced medical centers in China; however, finger-guided biopsy continues to be performed at certain primary hospitals. The present data demonstrates that TRUS-guided biopsy is superior when compared with finger-guided biopsy with regard to PCDR, particularly in patients with PSA ≤30 ng/ml or PV >46 cm^3^. Due to improvements in economic and health conditions, routine PSA screening and TRUS examinations have been introduced in elder males. Therefore, an increasing number of PCa patients have been detected in the early stage, and the overall PSA level has decreased. As a result of this, the use of TRUS-guided biopsy should be encouraged in developing countries, such as China. In certain advanced medical centers, MRI or other tools, such as elastography and contrast-enhanced TRUS, are also considered for assisting with biopsies.

Although novel biopsy tools and methods have been approved quickly, the optimal number of cores and distribution for conducting prostate biopsies remain controversial. Numerous studies proposed that the PCDR increases as the number of biopsy cores increases. Elabbady *et al* (12) reported that the 12-core biopsy increased the PCDR from 25.8% to 36.4% during a comparison with 6-core biopsy. Similarly, the PCDR was improved from 7.7% to 13.8% in the studies of Kojima *et al* (13) and Matsumoto *et al* (14). Certain studies showed different conclusions. In a randomized trial conducted by Naughton *et al* (8) no significant difference in PCDR between 6-core and 12-core biopsies was found. However, in the study by Kim *et al* (15), the PCDR of 12-core biopsy was identified to be lower than that of the 6-core biopsy (14.4% vs. 17.2%). In the current study, the lateral 6-core and 12-core biopsies were associated with a higher PCDR when compared with that of the medial 6-core biopsy. This may have been due to the prostate cancer predominantly occurring in the prostatic peripheral zone. The distribution of the cores in the present study were directed by TRUS and the results demonstrate that the distribution of biopsy cores is important when detecting the lesions. This may explain why certain studies found a significant improvement in the PCDR in cases where more biopsy cores were used, while other studies showed negative results. Notably, the 12-core biopsy did not demonstrate obvious superiority when compared with lateral 6-core biopsy, although the 12-core biopsy did exhibit a higher PCDR. This may be due to the positive rate of the medial 6-core biopsy, which reduced the difference in positive detection rates between the 12-core and lateral 6-core biopsies. Thus, the results identified the critical value of lateral biopsy cores in prostate cancer detection.

The PCDR of the entire 13-core biopsy was comparable with the 12-core biopsy, which appeared to demonstrate that the extra 13th core was insignificant. However, the entire 13-core biopsy was significantly superior with regard to PCDR, when compared with the lateral 6-core biopsy. During the biopsy procedures, particularly the 12-core biopsy, one or two cores were located in the areas that exhibited abnormal TRUS or MRI findings. This may explain the similar PCDR between the entire 13-core biopsy and the 12-core biopsy. Further analysis demonstrated that the positive rate of the extra 13th core, which was directed towards the hypoechoic lesions on the TRUS image or the abnormal MRI signal, was significantly higher than the mean positive rate of the systematic 12-core biopsy. This verified that the areas with abnormal TRUS or MRI findings exhibited a higher positive rate of cancer than other areas. Therefore, the systematic 12-core biopsy plus the extra 13th core biopsy was beneficial for improving the PCDR.

In conclusion, the positive rate of the extra 13th core, which was directed towards the abnormal MRI signal showed an insignificant superiority when compared with the hypoechoic lesions that were observed on the TRUS image. MRI has an obvious advantage over TRUS when detecting and observing prostate tissues. However, MRI was not used directly to guide the biopsy in the current study, which is a limitation of the study. Another limitation was the restricted number of cases included in the present study. Only 32 patients were included for the analysis, thus, an increased number of cases and the performance of genuine MRI-guided biopsies are required in future studies to evaluate the value of MRI in determining PCDR.

## Figures and Tables

**Table I:** Demographic and clinical characteristics of 2,707 patients.

	Prostate cancer detection rate		
		
Variable	Negative, n (%)	Positive, n (%)	P-value
PSA, ng/ml			<0.001
0–4	140 (87.0)	21 (13.0)	
4.01–10	664 (80.4)	162 (19.6)	
10.01–20	602 (72.8)	225 (27.2)	
20.01–30	182 (58.3)	130 (41.7)	
>30	140 (24.1)	441 (75.9)	
Age, years	68.3±8.12	71.1±7.12	0.008
fPSA, ng/ml	2.3±3.35	8.5±36.40	<0.001
PV, cm^3^	52.28±29.25	41.27±22.85	<0.001
f/t ratio	0.17±0.098	0.12±0.072	<0.001
PSAD, ng/ml/cm^3^	0.32±0.42	2.04±9.36	<0.001
DRE finding			<0.001
Negative	1525 (76.9)	457 (23.1)	
Positive	203 (28.0)	522 (72.0)	
Echo level			<0.001
Regular	730 (72.0)	284 (28.0)	
Irregular	998 (58.9)	695 (41.1)	
Hypoechoic			<0.001
Negative	1342 (77.3)	393 (22.7)	
Positive	386 (39.7)	586 (60.3)	
Microcalcification			<0.001
Negative	1282 (71.4)	513 (28.6)	
Positive	446 (48.9)	466 (51.1)	

PSA, prostate-specific antigen; fPSA, free PSA; PV, prostate volume; f/t ratio, fPSA divided by total PSA; PSAD, PSA density; DRE, digital rectal examination.

**Table II:** Prostate cancer detection rate of finger- and TRUS-guided biopsy stratified by PSA values and the PVs.

	Prostate cancer detection rate		
		
Variable	Negative, n (%)	Positive, n (%)	P-value
PSA, ng/ml			
0–30			<0.001
Finger-guided	996 (77.8)	284 (22.2)	
TRUS-guided	592 (70.0)	254 (30.0)	
>30			0.111
Finger-guided	86 (26.6)	237 (73.4)	
TRUS-guided	54 (20.9)	204 (79.1)	
PV, cm^3^			
0–46			0.336
Finger-guided	490 (55.7)	390 (44.3)	
TRUS-guided	379 (53.2)	334 (46.8)	
>46			<0.001
Finger-guided	592 (81.9)	131 (18.1)	
TRUS-guided	267 (68.3)	124 (31.7)	

PSA, prostate-specific antigen; TRUS, transrectal ultrasound; PV, prostate volume.

**Table III:** Prostate cancer detection rate of hypothetical medial 6-core, lateral 6-core, 12-core and entire 13-core biopsies guided by transrectal ultrasound.

	Prostate cancer detection rate		
		
Variable	Negative, n (%)	Positive, n (%)	P-value
Medial 6-core vs. lateral 6-core			0.020
Medial 6-core	749 (67.8)	355 (32.2)	
Lateral 6-core	696 (63.0)	408 (37.0)	
Medial 6-core vs. 12-core			<0.001
Medial 6-core	749 (67.8)	355 (32.2)	
12-core	655 (59.3)	449 (40.7)	
Lateral 6-core vs. 12-core			0.081
Lateral 6-core	696 (63.0)	408 (37.0)	
12-core	655 (59.3)	449 (40.7)	
Lateral 6-core vs. entire 13-core			0.033
Lateral 6-core	696 (63.0)	408 (37.0)	
Entire 13-core	646 (58.5)	458 (41.5)	
12-core vs. entire 13-core			0.729
12-core	655 (59.3)	449 (40.7)	
Entire 13-core	646 (58.5)	458 (41.5)	

**Table IV:** Positive rate of the 13th core that was directed towards abnormal TRUS or MRI findings and the mean positive rate of systematic 12-core biopsy in patients with confirmed prostate cancer.

	Prostate cancer detection rate		
		
Variable	Negative, n (%)	Positive, n (%)	P-value
TRUS-guided			<0.001
Mean positive rate	2387 (43.4)	3109 (56.6)	
Extra 13th core	44 (29.1)	107 (70.9)	
MRI-guided			0.006
Mean positive rate	2387 (43.4)	3109 (56.6)	
Extra 13th core	6 (18.8)	26 (81.2)	
TRUS vs. MRI			0.280
TRUS	44 (29.1)	107 (70.9)	
MRI	6 (18.8)	26 (81.2)	

TRUS, transrectal ultrasound; MRI, magnetic resonance imaging.
